# Malignant haemangiopericytomas of omentum presenting as left inguinal hernia: A case report

**DOI:** 10.1016/j.amsu.2021.01.070

**Published:** 2021-01-23

**Authors:** Sana shahid, Hina Khan, Muniba Mehmood, Khaled Abdullah Rage, Summaya Saeed

**Affiliations:** a Dr. Ruth K. M. Pfau Civil Hospital, Karachi, Pakistan; b Dow University of Health Sciences, Karachi, Pakistan

**Keywords:** Haemangiopericytomas, Omentum, Inguinal hernia, Case report, Solitary fibrous tumor

## Abstract

**Background:**

Hemangiopericytomas (HPC) are vascular tumors and can be found at any place where vessels are present. These were previously known as ‘extrapleural Solitary Fibrous Tumour’. These tumors may reoccur and metastasize after surgical excision. We present herein a HPC of the greater omentum, diagnosed as left inguinal hernia preoperatively.

**Case presentation:**

A 61-year-old male, presented with a huge painless mass in his left inguinoscrotal region secondary to weigh-lifting associated with malaise and vague abdominal pain. A well-defined, non-tender, and firm mass was found at the left lower abdomen extending to the left inguinoscrotal region. Based on the examination, a diagnosis of indirect inguinal hernia was made. Abdominal ultrasound showed a heterogeneous, hyporeflective, and vascularized mass. Contrast-enhanced computed tomography scan identified a localized, extraperitoneal, heterogeneously hypodense, well-defined, and lobulated mass, with marked contrast enhancement. On exploration, an encapsulated large mass originating from the omentum with enormously dilated blood vessels was excised. On histopathology, a neoplastic lesion, composed of spindle-shaped cells and moderate cytoplasm was identified. The blood vessels appeared thin-walled with a staghorn appearance in hemangiopericytic pattern. Omental sections showed fibro adipose tissue with dilated lymphatics and thick-walled blood vessels. Features were consistent with a malignant HPC of 20 × 14 × 10 cm.

**Conclusion:**

We present an unusual presentation of primary omental malignant HPC as an inguinal hernia, treated by complete surgical resection. These tumors are rare therefore, timely diagnosis is important for proper evaluation, diagnosis, and treatment. It also requires long-term follow up for better survival.

## Highlights

• Hemangiopericytoma (HPC), previously known as ‘extrapleural Solitary Fibrous Tumor’, is a vascular tumor.
• A well-defined, non-tender, and firm mass was found at the left lower abdomen extending to the left inguinoscrotal region.
• Based on the examination, a diagnosis of indirect inguinal hernia was made.
• CT scan showed a contrast enhancing, localized, extraperitoneal, heterogeneously hypodense, and lobulated mass.
• On exploration, an encapsulated large mass originating from the omentum with enormously dilated blood vessels was excised.
• On histopathology, features were consistent with a malignant HPC of 20 × 14 × 10 cm.

## 1 Introduction

Primary omental malignancies are extremely rare and difficult to diagnose. A solitary fibrous tumor (SFT) is a rare primary omental malignant tumor that usually arises from the pleura. Extrapleural SFT, that can be seen in the head and neck, abdomen, pelvis, or skin includes extra-meningeal SFTs, hemangiopericytoma, lipomatous hemangiopericytoma, and giant cell angiofibroma. These can be benign or malignant, with an overall incidence of 0.6%. These tumors are common in the 5th and 6th decade of life [[1], [2], [3]].

Hemangiopericytoma (HPC), previously defined by the World Health Organization as an extrapleural SFT, is a vascular tumor that is believed to arise from the pericytes of Zimmerman, showing evidence of myoid or myofibroblastic differentiation [1,4,5].

To the best of our knowledge, malignant hemangiopericytomas presenting as an inguinal hernia has not been reported before. There are 20 case reports about benign HPC but in literature, only four cases of malignant HPC have been reported [2,[6], [7], [8]]. Here, according to the SCARE criteria [9], we describe a rare case of malignant HPC arising from the omentum and presenting as left inguinal hernia.

## 2 Case presentation

**Fig. 1:**
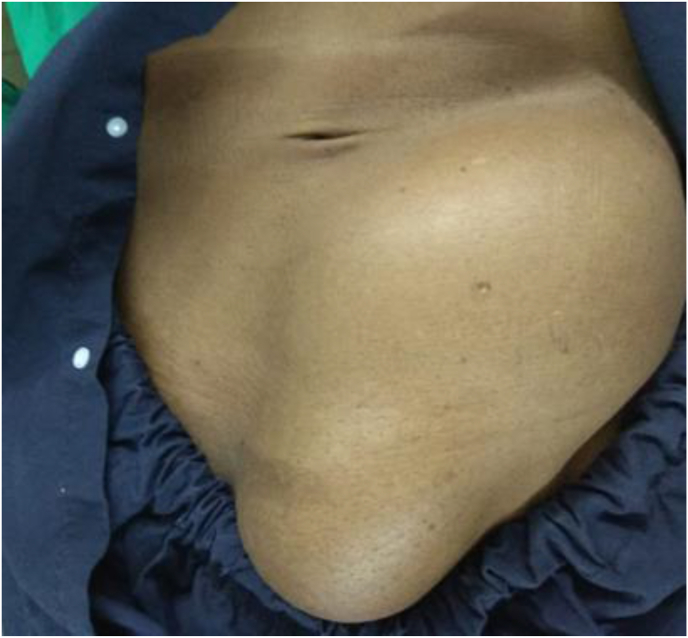
Inspection of the abdomen showing a mass extending from the left lower abdomen to the right inguinal region.

A 61-year-old male, with no known comorbid, presented with a huge painless mass in his left inguinoscrotal region associated with malaise and vague abdominal pain for one month. He had a history of lifting a Camel after which a lump gradually developed. On abdominal examination, he revealed a well-defined, non-tender, and firm mass of 15 × 10 cm at the left lower abdomen involving the left inguinal region as well as the left hemiscrotum (Fig. 1). Based on the examination, a diagnosis of indirect inguinal hernia was made.

**Fig. 2:**
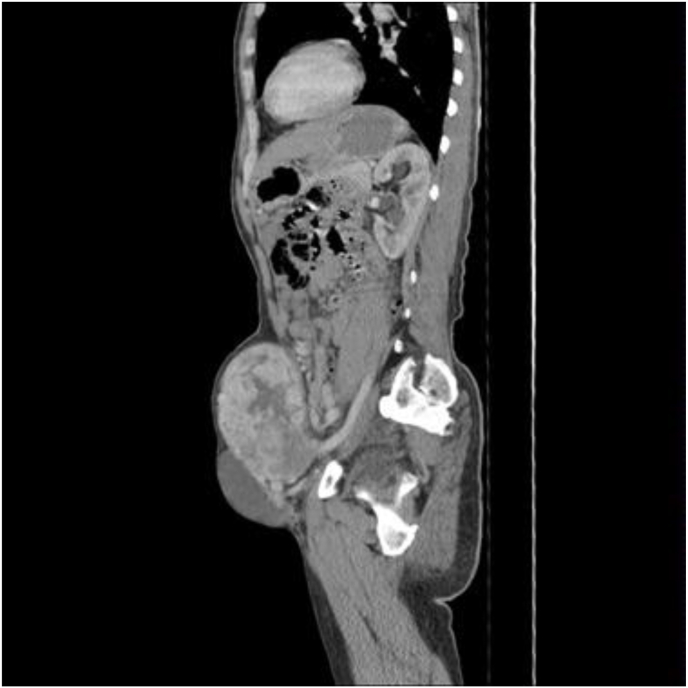
Sagittal view of a computed tomography scan showing a hypodense, heterogenous, extraperitoneal mass at the left iliac fossa, adjacent to the anterior abdomen.

The complete blood count and biochemical tests were within the reference values. Tumor markers i.e. Carcinoembryonic antigen, Alpha-fetoprotein, Carbohydrate antigen (CA) 19.9, and CA 125 were within range. Abdominal ultrasound showed a heterogeneous structure with hyporeflective areas and was highly vascularized. Contrast-enhanced computed tomography (CT) scan identified a mass at the left iliac fossa, adjacent to the anterior wall of the abdomen, but extraperitoneal, measuring 15 × 10 × 10 cm. The mass was heterogeneously hypodense, well-defined, and lobulated, with marked enhancement effect after contrast administration, comparable to vascular density, but no evidence of distant metastasis or lymphadenopathy (Fig. 2).

**Fig. 3:**
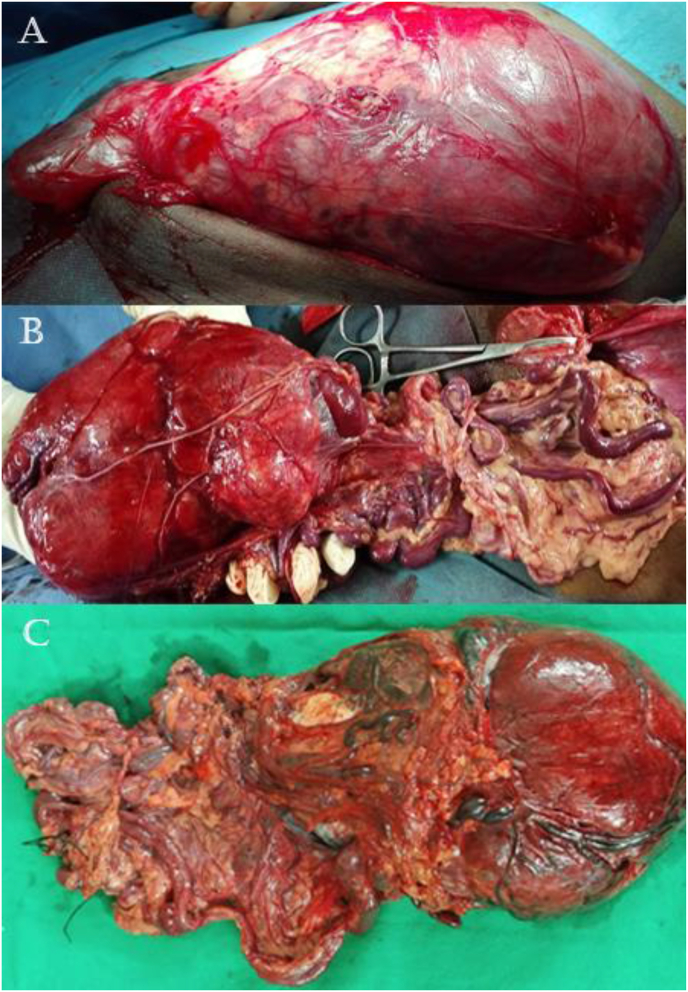
Perioperative presentations showing (A) an encapsulated mass, (B) a view of the mass taken out of the abdominal cavity, (C) gross presentation of the excised mass with dilated blood vessels and tumor-free surgical margins.

Surgery was planned under general anesthesia and the patient underwent an en-bloc local resection of the tumor via an inguinal approach. An inguinal crease incision was given over the swelling. On exploration, we found an encapsulated large mass originating from the omentum with enormously dilated blood vessels and a 5 × 5 cm hydrocele, which was excised and weighed around 1.5 kg. Testis was unremarkable (Fig. 3A and B). The macroscopic surgical margin was tumor-free.

On gross examination, an irregular dark brown to grey-white mass with prominent dilated vessels on the surface was seen. On sectioning, the cut surface of the mass was light brown with cystically dilated hemorrhagic spaces and separately lying omental tissue piece with prominent congested and dilated vessels measuring 17 × 17 × 5 cm. Microscopically a neoplastic lesion was visible, composed of spindle-shaped cells having moderate cytoplasm and bland nuclei without necrosis. The blood vessels appeared thin-walled with a staghorn appearance in hemangiopericytic pattern. Few mitotic figures were seen. No testicular parenchyma or epididymal tissue was reported. Omental sections showed fibro adipose tissue with dilated lymphatics and thick-walled blood vessels. Features were consistent with malignant HPC of 20 × 14 × 10 cm (Fig. 3C).

Immunohistochemical analysis showed strongly positive CD34 and patchy positive STAT 6 biomarkers while it was negative for cytokeratin, S100, ASMA, desmin, TLE-1, ER/PR, and EMA.

The patient was discharged on 3rd postoperative day without any complications. His recovery after surgery was satisfactory. At 12-months follow-up, there was no evidence of recurrence or metastasis and he is alive and well.

## 3 Discussion

Primary benign HPC of omentum represents <2% of all soft tissue tumors and malignant HPCs represent <1% of all vascular tumors [4,10]^.^ There are only four case reports (Table 1) of malignant HPC in the current literature [2,[6], [7], [8]].

**Table 1:** Review of malignant hemangiopericytoma case reports.

Case	Age (years)	Size (cm)	Mitotic figures (/10HPF)	Recurrence/metastasis	Disease-free follow up (months)
**1**	60	24 × 19 × 10	25	Absent	4
**2**	40	9	9	Present	34
**3**	29	28 × 25 × 11	0	Absent	48
**4**	57	18 × 11 × 6	6	Absent	32
**Our Patient**	61	17 × 17 × 5	Few	Absent	12

Immunochemistry is not always diagnostic of HPC however, cell markers of normal pericytes, such as desmin and actin, are not usually found in HPC cells [5]. They express CD34 (100%), CD99 and Bcl-2 (70–90%) antigens [1,4,11]. STAT6 is a reliable diagnostic immunohistochemical marker [12]. In our patient, CD34 and STAT 6 were present.

Besides a detailed physical examination and laboratory testing, detailed imaging investigations should be performed for the diagnosis of HPC [13]. Magnetic resonance imaging (MRI) can help in determining the extent of the tumor and its relationship with the surrounding structures better than the CT scan. Here in our case, the surgeon went for surgery after seeing the findings of the CT scan since surgery was required whatsoever due to the size of the lesion. Moreover, with our hospital already low on resources and patients low on affordability, the need for an extra investigation was minimized.

HPC is a tumor of vascular origin, for which a combination of surgery, chemotherapy, and radiotherapy is required. En-bloc surgical excision has been the cornerstone of treatment [14]. Radiotherapy is preferred in inoperable cases and as an adjuvant treatment. However, the literature shows little evidence to support adjuvant chemotherapy [10,15,16]. Here, it was totally under the surgeon's discretion to operate.

For accurate diagnosis and effectiveness of treatment, CT or ultrasound-guided biopsy and immunohistochemical staining should be performed preoperatively. This not only helps in the definitive diagnosis but also helps in knowing the extent and prognosis of the mass lesion [13,17,18]. Latter was done here in this case while the former was not available in the hospital. Moreover, being vascular, pre-operative vascular embolization of these lesions can reduce the chances of bleeding during surgery [10,16].

## 4 Conclusion

We present an unusual presentation of primary omental malignant HPC as an inguinal hernia, treated by complete surgical resection. These tumors are rare therefore, timely diagnosis is important for proper evaluation, diagnosis, and treatment. It also requires long-term follow up for better survival.

## Consent of patient

Written informed consent was obtained from the patient for publication of this case report and accompanying images. A copy of the written consent is available for review by the Editor-in-Chief of this journal on request.

## Availability of data and material

All the available data is presented.

## Competing interests

None to declare.

## Funding

None.

## Disclaimer

The authors have no financial or proprietary interest in the subject matter of this article.

## Ethical approval

Ethical approval was acquired from the involved institution i.e. Civil Hospital, to collect the patient details, and present it as a publication.

## Sources of funding

None.

## Author contribution

Since there is no data analysis here, All authors i.e. Sana Shahid, Hina Khan, Muniba Mehmood, Khaled Abdullah Rage, and Summaya Saeed together did the concept and design of the study; data acquisition; and drafting of the manuscript. All authors critically revised the manuscript, approved the final version to be published, and agree to be accountable for all aspects of the work.

## Registration of research studies

Not applicable, since this is a case report.

## Guarantor

Muniba Mehmood.

## Declaration of competing interest

None.
